# Statin Use Can Attenuate the Decline in Left Ventricular Ejection Fraction and the Incidence of Cardiomyopathy in Cardiotoxic Chemotherapy Recipients: A Systematic Review and Meta-Analysis

**DOI:** 10.3390/jcm10163731

**Published:** 2021-08-22

**Authors:** Jean Kim, Yoshito Nishimura, Jakrin Kewcharoen, James Yess

**Affiliations:** Department of Internal Medicine, University of Hawai’i, 1356 Lusitana Str., Room 715, Honolulu, HI 96813, USA; nishimura-yoshito@okayama-u.ac.jp (Y.N.); jakrin@hawaii.edu (J.K.); jyess@queens.org (J.Y.)

**Keywords:** chemotherapy-induced cardiomyopathy, statin, chemotherapy, cardiotoxicity, left ventricular dysfunction, heart failure, cardio-oncology

## Abstract

There is insufficient evidence about the cardioprotective effects of statins against chemotherapy-induced cardiomyopathy. The MEDLINE and EMBASE databases were searched from inception to March 2021 for studies that reported the mean left ventricular ejection fraction (LVEF) before and after chemotherapy and the incidence of chemotherapy-induced cardiotoxicity in patients who received concurrent statin therapy and those who received chemotherapy alone. A random effects meta-analysis was performed to obtain the pooled weighted mean difference (WMD) and the 95% confidence interval (CI) for the mean final LVEF and the mean LVEF change, and the pooled odds ratio (OR) and the 95% CI of the incidence of chemotherapy-induced cardiomyopathy. Seven studies with 3042 patients were included in this meta-analysis (statin group: 1382 patients received concurrent statin with chemotherapy; control group: 1660 patients received chemotherapy alone). Patients in the control group had a more significant decline in LVEF (WMD = −6.08%, 95% CI: −8.55 to −3.61, *p* < 0.001) compared to those in the statin group. Additionally, the statin group had a significantly lower incidence of chemotherapy-induced cardiomyopathy compared to the control group (OR = 0.41, 95% CI = 0.28–0.60, *p* < 0.001). Consequently, our study showed a significant reduction in the incidence of chemotherapy-induced cardiomyopathy and the degree of LVEF decline in patients in the statin group compared to those in the control group.

## 1. Introduction

Cancer is one of the leading causes of mortality worldwide, with 9.9 million cancer-related deaths reported in 2020 [1,2,3,4]. Successful cancer treatments are on the rise, and so cancer survivorship continues to increase. Although chemotherapy has remained as one of the essential cancer treatment measures, various adverse effects have been noted, including chemotherapy-induced cardiotoxicity (CIC), which is characterized by a progressive decline in the left ventricular ejection fraction (LVEF) and subsequent heart failure, either in a reversible, stress-induced fashion [5,6,7] or in an irreversible manner that can be fatal and/or chronic [8,9,10].

The incidence of CIC is estimated to be around 1–5% of all cancer patients, and it typically occurs in a dose-dependent fashion [11,12,13]. In particular, patients receiving anthracyclines such as doxorubicin and HER2/neu receptor monoclonal antibodies including trastuzumab are more at risk of developing CIC [14]. Importantly, patients who develop CIC have been shown to have up to 3.5 times higher risk of mortality than those with cardiomyopathy from other causes. It may be because those with CIC often have sub-clinical disease progression in the early stages, with overt changes in symptoms only after they have sustained a significant level of cardiac damage [13], and limited preventive and treatment options are available, such as beta-blockers, angiotensin-converting enzyme inhibitors (ACEIs), and angiotensin receptor blockers (ARBs) [15,16].

Some individual studies have suggested that statins may also play an important role in decreasing the risk of CIC [17,18,19,20,21,22,23]. To the best of our knowledge, there is no meta-analysis that studied the overall pooled cardioprotective effect of statins against CIC. In this study, we employed a random-effects model for meta-analysis and sought to investigate the pooled effect for the difference in the degree of decline in LVEF and for the incidence of chemotherapy-induced cardiomyopathy in patients in the statin group, who received the concurrent statin during the chemotherapy, compared to those in the control group, who received chemotherapy only.

## 2. Materials and Methods

### 2.1. Search Strategy

A search for published studies indexed in the MEDLINE and EMBASE databases from inception to March 2021 was conducted using a search strategy that included the terms “statin”, “chemotherapy”, “cardiomyopathy”, and “heart failure”. The study included patients with all disease statuses and methods of conditioning regimens. There was no restriction based on the patients’ age, ethnicity, race, data sources, or study location. There was also no restriction based on the chemotherapy or the type or stage of cancer. Review articles, case reports, letters, commentaries, and studies in languages other than English were excluded. A manual search for additional pertinent studies or review articles using references from the retrieved articles was also completed.

### 2.2. Study Inclusion Criteria

The eligibility criteria for inclusion of studies are the following:

(1) Randomized controlled trials (RCTs), case-control studies, cohort studies (prospective or retrospective), and cross-sectional studies that reported changes in LVEF, the pre- and post-chemotherapy LVEF, and/or the incidence of new-onset heart failure (HF) in patients who were and were not receiving the concurrent statin during chemotherapy;
(2) Statistics such as odds ratio (OR), risk ratio (RR), hazard ratio (HR), or weighted mean difference (WMD) and its corresponding 95% confidence intervals (95% CI) and *p*-values or sufficient raw data for these calculations had to be provided.

### 2.3. Quality Assessment of the Included Studies

The Newcastle–Ottawa quality assessment scale (NOS), ranging from 0 to 9, was used to evaluate each study in three domains: recruitment and selection of the participants, similarity and comparability between the groups, and ascertainment of the outcome of interest among cohort studies. The Cochrane Collaboration tool for assessing risk of bias was used to evaluate the quality of each randomized controlled trial by assigning a score (high, low, or unclear) to each individual element from five domains (selection, performance, attrition, reporting, and other) [24].

### 2.4. Definition of Chemotherapy-Induced Cardiotoxicity

Chemotherapy-induced cardiotoxicity (CIC) is a heterogenous term that describes cardiotoxic effects from cancer therapeutics and encompasses mild asymptomatic myocardial injury and symptomatic heart failure with a decline in LVEF. The term chemotherapy-induced cardiotoxicity is often interchangeably used with chemotherapy-induced cardiomyopathy. Different criteria for diagnosis and surveillance of CIC have been suggested based on the type of chemotherapy. According to the expert consensus of the American Society of Echocardiography and the European Association of Cardiovascular Imaging, CIC is defined as a decrease in LVEF of over 10% below the lower limit of normal. In the studies included in our meta-analysis, the definition of CIC varied and includes incident HF with or without symptoms, hospitalizations or ED visits for HF, or LVEF <50–55% during the follow-up period of chemotherapy without evidence of heart failure before chemotherapy or other evident causes of cardiomyopathy as ascertained by the enrolling physicians.

### 2.5. Data Extraction

A standardized data collection form was used to obtain the following information from each study: title, name of authors, year of publication, country of origin, the number of participants in the statin therapy group and the control (no statin) group who underwent chemotherapy, and information about the change LVEF or the incidence of cardiomyopathy in the two groups before and after chemotherapy. In addition, information about the type of statins, cancer, chemotherapy, and mean follow-up duration was collected.

### 2.6. Statistical Analysis

Meta-analysis of the included studies was performed to determine the pooled effect size with a 95% CI. Two outcomes were of interest in the statin versus the (non-statin) control group receiving chemotherapy: (1) WMD of mean final LVEF after chemotherapy; (2) WMD of mean LVEF difference between pre- and post-chemotherapy (i.e., mean LVEF change); (3) the incidence of CIC after chemotherapy. In the studies that did not report the values of OR for the CIC incidence and its corresponding 95% CI, the number of subjects for the following were used to manually calculate the OR: (a) number of patients that had CIC after chemotherapy in the statin group, (b) number of patients without CIC after chemotherapy in the statin group, (c) number of patients with CIC after chemotherapy in the control group (no statin), and (d) number of patients without CIC after chemotherapy in the control group. Then, by using these provided data, OR and its corresponding 95% CIs were manually calculated. The heterogeneity of effect size estimates across the studies was quantified using the Q-statistic and the corresponding *p*-value or equivalent using the Higgins I-squared (I^2^) statistic. In our study, the meta-analysis was performed using the random-effects model, and the main results were summarized in the forest plots. To test the robustness of the results, a sensitivity analysis was conducted by performing meta-analyses excluding one study at a time. All analyses were performed using STATA 16 software (StataCorp LLC, College Station, TX, USA).

## 3. Results

### 3.1. Study Search Results

**Figure 1:**
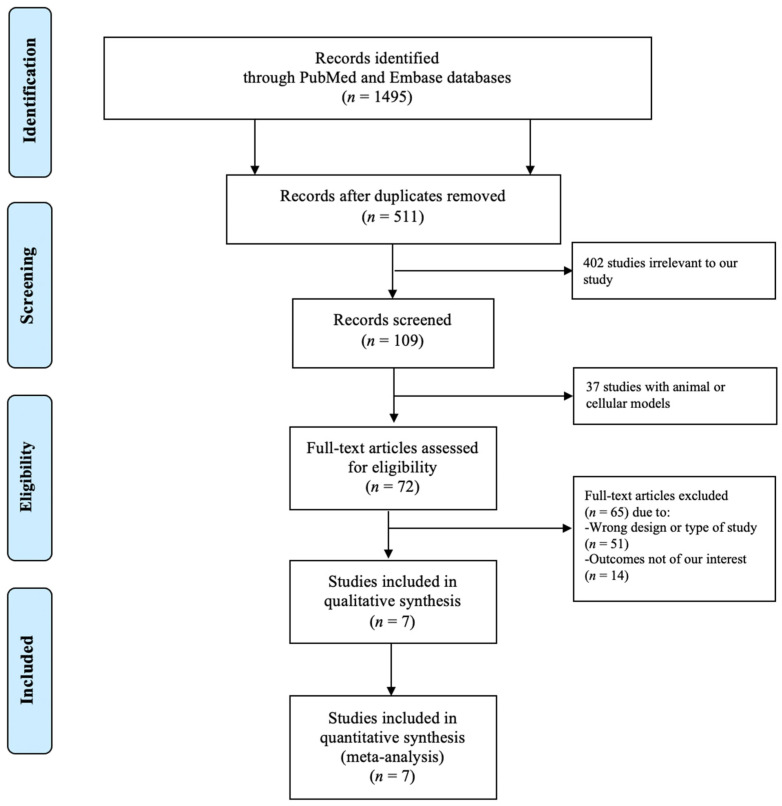
PRISMA (Preferred Reporting Items for Systematic Reviews and Meta-Analyses) diagram depicting the search methodology and selection process.

Figure 1 shows a PRISMA (Preferred Reporting Items for Systematic Reviews and Meta-Analyses) flow diagram that depicts the process of identification, screening, eligibility, and inclusion or exclusion of the studies. The initial search of the PubMed and EMBASE databases yielded 1495 articles. A total of 511 duplicate studies were removed, followed by elimination of 402 studies that were irrelevant to our study and 37 studies with animal or cellular models. Subsequently, 72 studies underwent title and abstract review. Of these articles, 51 studies were excluded because they were not of the appropriate type or design of study for our analysis, and 14 studies were eliminated as they did not have the outcomes of interest. The final analysis included seven unique studies with a total of 3042 subjects.

### 3.2. Description of the Included Studies and Quality Assessment

A total of seven studies with 3042 subjects (1382 subjects received the concurrent statin) were included in our meta-analysis [17,18,19,20,21,22,23]. The main characteristics of the included studies are described in Table 1. Regarding the study design, five studies were observational and two were RCTs. The mean pre- and post-chemotherapy LVEF information was provided in the studies by Nabati et al. [21], Acar et al. [18], Calvillo-Argüelles et al. [19], and Chotenimitkhun et al. [20]. In regards to the incidence of cardiomyopathy, the study by Calvillo-Argüelles et al. [19] and Abdel-Qadir et al. [17] provided the value of OR and its corresponding 95% CI. In the studies by Acar et al. [18], Seicean et al. [22], and Tase et al. [23], the raw data for the number of subjects with CIC in the control and the statin groups are provided, and calculations were manually performed to obtain the OR and its corresponding 95% CI. NOS of the five selected studies ranged from 6 to 9 with a mean score of 8, reflecting a high quality of these studies. For the two RCTs included in the meta-analysis, the Cochrane Collaboration tool for assessing the risk of bias was used. This showed a low risk of bias in most categories, except for the lack of blinding in the study by Acar et al. [18].

### 3.3. Quantitative Meta-Analysis Results

**Figure 2:**
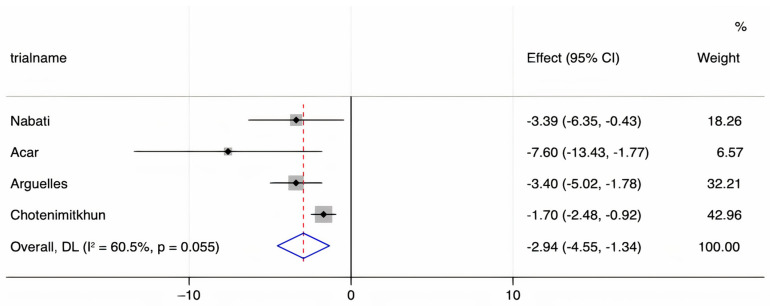
A forest plot demonstrating the WMD (weighted mean difference) for the mean final LVEF (left ventricular ejection fraction) post-chemotherapy in patients (who received chemotherapy alone) in the control group compared to patients who received concurrent statin in the statin group. Weights are from the random-effects model.

**Figure 3:**
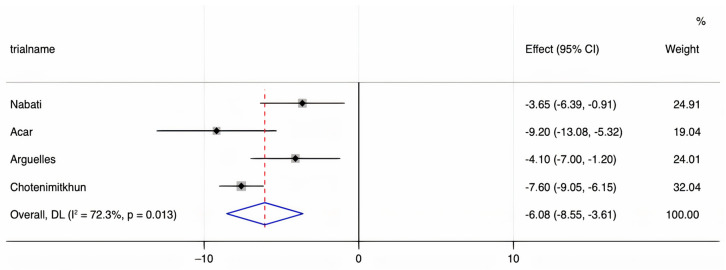
A forest plot demonstrating the WMD (weighted mean difference) for the mean LVEF (left ventricular ejection fraction) change before and after chemotherapy in patients (who received chemotherapy alone) in the control group compared to patients who received concurrent statin in the statin group. Weights are from the random-effects model.

**Figure 4:**
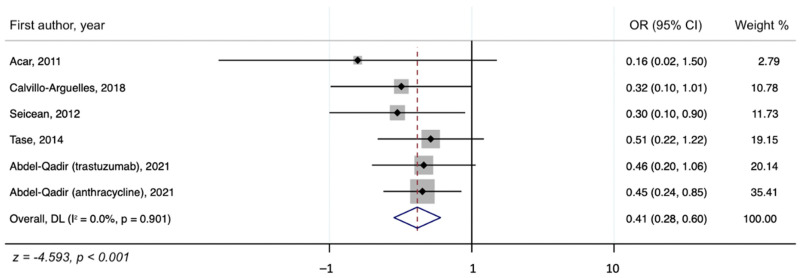
A forest plot showing the OR (odds ratio) for the association of concurrent statin use with chemotherapy and the incidence of chemotherapy-induced cardiomyopathy.

We assessed the presence of heterogeneity among the studies in terms of the Q-statistic and the corresponding *p*-value. In the present case of *p* < 0.1, heterogeneity among studies existed. We also quantified the heterogeneity degree by using the I2 statistic which showed substantial heterogeneity among studies (I2 > 60%). Thus, in our study, we employed the random-effects model to analyze the pooled effect size. The mean final post-chemotherapy LVEF in the control group (without concurrent statin use during chemotherapy) was significantly lower than that in the statin group (with concurrent statin use during chemotherapy) (WMD = −2.94%, 95% CI: −4.55–−1.34, *p* < 0.001). Additionally, patients in the control group had a more statistically significant mean LVEF change (i.e., mean LVEF difference between pre- and post-chemotherapy) compared to patients in the statin group (WMD = −6.08%, 95% CI: −8.55–−3.61, *p* < 0.001). Finally, it was found that the intervention group receiving statin had a significantly lower incidence of chemotherapy-induced cardiomyopathy compared to the control group (OR = 0.41, 95% CI = 0.28–0.60, *p* < 0.001). The forest plots demonstrating the WMD of the final LVEF post-chemotherapy, the WMD of the change in LVEF between pre- and post-chemotherapy, and the OR for the incidence of chemotherapy-induced cardiomyopathy are shown in Figure 2, Figure 3 and Figure 4, respectively.

### 3.4. Publication Bias

We aimed to investigate potential publication bias via the funnel plot and Egger’s test [25,26]. However, as we only had up to seven studies in the analysis, this number was insufficient to reject the assumption of no funnel plot asymmetry; thus, we did not perform a funnel plot or Egger’s test.

### 3.5. Sensitivity Analysis

To examine the robustness of the pooled OR and 95% CI in the whole group, sensitivity analyses were undertaken by excluding one individual study at a time and they showed no significant changes, suggesting that our results are robust.

## 4. Discussion

The present study is the most up-to-date systematic review and meta-analysis on the preventive effects of statins against chemotherapy-induced cardiomyopathy. Our results underscore that statins may provide a significant preventive benefit against CIC, especially for those who received anthracyclines and trastuzumab. We found that patients in the control group who did not receive statins with chemotherapy had a more significant decline in LVEF compared to those of the statin group (WMD = −6.08%, 95% CI: −8.55 to −3.61, *p* < 0.001). In addition, those who received concurrent statins in the statin group had lower odds of developing CIC compared to the control group (OR = 0.41, 95% CI = 0.28–0.60, *p* < 0.001). These results suggest that statins are promising cardioprotective agents against CIC.

Interestingly, those who received statins were more likely to have cardiovascular risk factors, including diabetes mellitus, hypertension, or coronary artery disease, as reported by Carvillo-Argüelles et al., Chotenimitkhun et al., and Seicean et al. Nevertheless, the authors found that statin use was independently associated with a reduced occurrence of CIC after adjustment for these risk factors. In addition, Abdel-Qadir et al. demonstrated that those who received statins had a lower risk of CIC in their sensitivity analyses, removing those who had interim acute myocardial infarction with imputation of the elevated low-density lipoprotein levels. The results suggested that the protective mechanism of statins may be independent of their cholesterol-lowering effects. Although the exact pathophysiology of CIC remains unclear, it is proposed that drugs such as anthracyclines increase the production of oxygen-derived free radicals in cardiac myocytes and increase the intracellular anthracycline-iron complex accumulation [14,27,28,29,30], leading to increased oxidative stress and subsequent necrosis of the cells. Statins, or hydroxymethylglutaryl-CoA (HMG-CoA) reductase inhibitors, which are cholesterol-lowering drugs primarily used for primary and secondary prevention of cardiovascular diseases, have also been shown to render prophylactic effects against CIC via their action of reducing oxidative stress at the cellular level [31,32].

Although cardiac dysfunction related to chemotherapy could be addressed with an interruption or discontinuation of chemotherapy, the cessation of chemotherapy in cancer patients may be related to poor clinical outcomes from the oncology standpoint. Furthermore, 0.5–2.5% of patients with chemotherapy-induced cardiomyopathy may have end-stage heart failure requiring a left-ventricular assist device or even heart transplant [9,10,33,34,35]. Due to the possible poor trajectory, guidelines for prevention and surveillance of chemotherapy-induced cardiomyopathy are imperative. Several trials assessing the efficacy of statins in preventing CIC are underway, including a trial investigating the effect of atorvastatin in the preservation of LVEF 24 months after initiation of anthracycline-based adjuvant therapy for breast cancer patients in the National Institutes of Health (NIH) sponsored study PREVENT (Preventing Anthracycline Cardiovascular Toxicity with Statins) [36].

Several limitations in the current study should be noted. First, two RCTs were included in our review, while the others were non-randomized experimental trials and a cohort study involving those who received statins either for primary or secondary prevention. This resulted in an increased heterogeneity and a smaller number of participants. Additionally, the selected studies investigated different types of statins with different chemotherapy and cancers, further contributing to the heterogeneity of the studies. However, the random-effects model was employed in our meta-analysis to account for this heterogeneity, and ultimately, the sensitivity analyses showed that our results were robust. Secondly, Egger’s test could not be performed, as fewer than 10 studies were included in the meta-analysis, so publication bias cannot be ruled out. Thirdly, the statin group was more likely to receive other potential cardioprotective agents such as beta-blockers or renin-angiotensin system inhibitors than the control group due to their cardiovascular comorbidities. This can be viewed as a possible confounder. In addition to the cardiovascular medications, there are several other potential confounders such as age, gender, race, body mass index (BMI), lipid level, and cardiovascular risk factors (diabetes mellitus, hypertension, coronary artery disease). However, adjustments for the baseline LVEF and potential confounders were made in the included studies [17,19,20,22], and, after adjustments, the protective effects of statins (e.g., differences in the final LVEF and the LVEF change between the statin and the control groups) remained statistically significant. Moreover, there is still no clear consensus in the current cardio-oncology guidelines about the significance of the potential protective effects of beta-blocker and angiotensin system inhibitors for CIC. Lastly, the included studies had an average follow-up period of 21.5 months, and the long-term preventive effects of statins against chemotherapy-induced cardiomyopathy are still unclear. Despite these limitations, the study presents promising evidence that statins may provide significant cardioprotective effects for those receiving cardiotoxic chemotherapy, and further investigation into the role of statins against CIC is important in this regard.

## 5. Conclusions

In conclusion, via meta-analysis, statins were found to have a cardioprotective effect against chemotherapy-induced cardiomyopathy. Specifically, the control group, which did not receive statins, had a more significant decline in LVEF after chemotherapy, with a WMD of −6.08% (95% CI: −8.55–−3.61, *p* < 0.001), compared to the statin group. Additionally, compared to the control group, the statin group had a significantly lower incidence of chemotherapy-induced cardiomyopathy (OR = 0.41, 95% CI = 0.28–0.60, *p* < 0.001). Further, a larger-scale RCT with extended follow-up period is needed to corroborate our findings.

## Figures and Tables

**Table 1:** Main characteristics of the included studies.

Author	Country	Published Year	Study Type	Number of Participants (*n* = 3042)	Mean or Median Age (Years)	Mean Follow-Up Durations (Months)	Cancer	Chemotherapy	Statin, *n* (*n* = 1382)
Acar	Turkey	2011	RCT	40	53.0 ± 15.0	6	NHL MM Leukemia	Anthracycline	Atorvastatin, 20
Calvillo- Argüelles	Canada	2018	Case control	129	62.0 ± 9.0	11	Breast	Trastuzumab	Atorvastatin, 24 Rosuvastatin, 11 Simvastatin, 5 Pravastatin, 3
Chotenimtkhun	U.S.	2013	Cohort	51	48.0 ± 2.0	6	Breast Leukemia Lymphoma	Anthracycline	Atorvastatin, 5 Simvastatin, 9
Nabati	Iran	2018	RCT	77	49.3 ± 11.2	6	Breast	Anthracycline Trastuzumab	Rosuvastatin, 38
Seicean	U.S.	2012	Cohort	201	51.5 ± 10.8	31.2	Breast	Anthracycline	N/A, 67
Tase	Romania	2014	Cohort	432	57.5 ± 11.2	30.6	Gastric	Anthracycline	Rosuvastatin, 77 Atorvastatin, 52 Other, 15
Abdel-Qadir	Canada	2021	Cohort	2112	69.0 (IQR 67–72) *	60	Breast	Anthracycline Trastuzumab	Rosuvastatin, 491 Atorvastatin, 433 Simvastatin, 82 Pravastatin, 27 Other, 23

Abbreviations: IQR, interquartile range; N/A, not applicable; RCT, randomized control trial. In the last column, “*n*” represents the number of patients who received statin. * Median age of patients who received anthracyclines. Median age for the trastuzumab group is 71 years (IQR: 68–75).

## Data Availability

The datasets generated and analyzed during the current study are available from the corresponding author on reasonable request.
